# Personalized medicine: when the common becomes the rare

**DOI:** 10.3389/fmed.2024.1523594

**Published:** 2024-12-18

**Authors:** Daniel J. O'Connor

**Affiliations:** The Association of the British Pharmaceutical Industry (ABPI), London, United Kingdom

**Keywords:** personalized medicine, small population research, innovative trial design, regulatory science, rare disease

## Introduction

Historically, much of cancer drug development was predominantly characterized by traditionally described common anatomical tumors with the ability to conduct large well-powered randomized clinical trials. The emergence of personalized medicine as a discipline and tool, often defined as using characterization of individuals' phenotypes and genotypes for tailoring the right therapeutic strategy for the right person at the right time, has created a new paradigm that offers clear advantages for patients in terms of their treatment choices but coupled with some intrinsic challenges. The personalized medicine approach provides opportunities to maximize the benefits of our increasing understanding of the molecular sciences, diagnostic capabilities, better clinicopathological correlation, and enhanced abilities in pharmacological intervention. However, the consequence of more targeted approaches in drug development for the trial population and clinical programme means it is often harder to find patients to recruit to trials, there are less patients overall to gather data in each subset from the broader condition, there is more complexity in the development of novel products and there is a need to implement innovative approaches for successful evidence generation (1). This Opinion article reflects on some of the impacts in this space with a focus on the need to address this shifting and growing paradigm in oncology of when the common becomes the rare.

## Impact of the common becoming the rare

The impact of an increasingly personalized and targeted approach to drug development in oncology are multiple but in summary include the need to:

- Ensure diagnostic consistency, efficiency and capacity both at the clinical trial level and in the health system, with the additional complexity for the sponsor to have to navigate companion diagnostic or *in vitro* diagnostic requirements and regulations (2).
- Demonstrate that the targetable characteristics defining the subset have a plausible association to the tumor, and is linked in a way that the absence of these characteristics will render the medicinal product ineffective in the broader population. This allows for predictive enrichment strategies, where the development programme selects subjects who are more likely to respond to a treatment. This helps to reduce the heterogeneity of the study population and enhances the power of the trial.
- Capture and interpret natural history data in patients with specific pathogenic variants in order to better understand prognostic and therapeutic outcomes and determine therapeutic windows, surrogate endpoints and monitor patient outcomes.
- Understand the broader regulatory and incentive impact including taking into account the varying views in the global regulatory system as to whether a rare subset or a histology independent indication is considered as valid for orphan drug designation, labeling or indication aspects and ability to use expedited and/or flexible drug licensing pathways (4).
- Determine the opportunities to utilize the benefits of medicines derived from platform technologies (a well-understood and reproducible technology which can be adapted for more than one medicine sharing common structural elements and a standardized manufacturing process).
- Factor in smaller populations of patients when making return on investment or commercial decisions.
- Acknowledge and address how smaller subsets of patients will impact the clinical programme and trial design, patient recruitment and center choice, and how the evidence generation can fit with the expectations of decision makers such as regulators and payers.

## Subsetting the common

The increasing subsetting of common cancers into rarer cohorts is one of the most impactful aspects of personalized medicine in terms of the nature of the clinical development programme in oncology, particularly for the choice of trial design from a feasibility, scientific or ethical perspective and the balance between what data might be collected pre-market and post-market. Such small population research requires compromise, and clinical development programmes which involve large numbers of patients are increasingly not possible. Key to keep in mind is the objective of generating the best evidence base through rigorous planning, exploring with regulators the acceptability of novel and innovative methodology and approaches (e.g., basket study, adaptive design, digital twin, and synthetic data) and making often difficult decisions in terms of the overall strategic approach (e.g., uncertainties in the interpretation of single arm study, utility of under-powered randomized approaches). As always, the requirement for statistical efficiency should be balanced against the need for drawing clinically relevant and scientifically robust conclusions where the total number of eligible subjects may be very limited. An acknowledged concern is that smaller studies are likely more susceptible to the effects of variability, and missing data will have a greater impact on the study conclusions, where policy makers, including medicines regulators and medicines payers, have to make decisions with less data, which can imply decisions are made with a greater degree of uncertainty. Ultimately smaller pre-market exposure will increasingly equate with the increased importance of and emphasis on post-market real world data collection from efficacy and safety perspectives. Although there is great interest in the potential utility of real world evidence, views on the right approach and application are still evolving, meaning that it is essential that development programmes are prospectively discussed with regulators and payers where these mechanisms exist (5).

## Discussion

Personalized medicine represents the best approach for an optimized benefit risk balance for patients. These approaches are revolutionizing drug development in oncology (and elsewhere), where once common conditions are subsetted into smaller cohorts or even therapies for the “individualized” patient (3). The rise of small population research is shifting the traditional paradigm of drug development with a need to utilize extrapolation methods, innovative trial designs and digital solutions, real world data collection and flexible regulatory and access approval systems. Innovative trial approaches such as basket type designs may help drive efficiencies and allow “scaling up,” with the added benefit for inclusion of rare cancer patients who may in the past have been neglected (6).

As an evolving scientific and policy area, we need to work together to ensure better system alignment on evidence generation approaches, and foster pragmatic and flexible regulatory and access pathways that acknowledge the challenges, helping to ensure that patients do not miss out on twenty-first century science advances.
